# Vacancy assisted diffusion on single‐atom surface alloys

**DOI:** 10.1002/cphc.202000838

**Published:** 2020-12-03

**Authors:** David Mahlberg, Axel Groß

**Affiliations:** ^1^ Institute of Theoretical Chemistry Ulm University 89069 Ulm Germany; ^2^ Institute of Theoretical Chemistry Ulm University 89069 Ulm Germany; ^3^ Helmholtz Institute Ulm (HIU), Electrochemical Energy Storage 89069 Ulm Germany

**Keywords:** Bimetallic surfaces, Density functional theory calculations, Diffusion, Kinetic Monte Carlo Simulations, Vacancies

## Abstract

Bimetallic surfaces can exhibit an improved catalytic activity through tailoring the concentration and/or the arrangement of the two metallic components. However, in order to be catalytically active, the active bimetallic surface structure has to be stable under operating conditions. Typically, structural changes in metals occur via vacancy diffusion. Based on the first‐principles determination of formation energies and diffusion barriers we have performed kinetic Monte‐Carlo (kMC) simulations to analyse the (meta‐)stability of PtRu/Ru(0001), AgPd/Pd(111), PtAu/Au(111) and InCu/Cu(100) surface alloys. In a first step, here we consider single‐atom alloys together with one vacancy per simulation cell. We will present results of the time evolution of these structures and analyse them in terms of the interaction between the constituents of the bimetallic surface.

## Graphical abstract

**Vacancies matter**: metal atoms can typically only change places in metals through vacancy‐assisted diffusion. Here we address the diffusion in single‐atom surface alloys by performing kinetic Monte Carlo simulations based on density functional theory calculations. We illustrate how the separation and recombination with a surface vacancy determine the trajectory of the foreign atom in such a surface alloy.

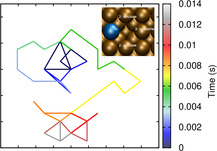

## 1 Introduction

Catalysts play a significant role when it comes to improving the efficiency and selectivity of a chemical reaction. It is well‐known that the activity and selectivity of bimetallic catalysts is often superior to the one of elemental metals due to the additional degrees of freedom through the variation of the concentration and/or the arrangement of the two metallic components.[^1^, ^2^, ^3^, ^4^] Furthermore, the replacement of a rare and expensive metal by a more abundant and less expensive metal can lead to the reduction of costs and a higher sustainability of the catalyst.[^5^, ^6^] The catalytic properties of bimetallic surfaces can be rather reliably assessed using first‐principles electronic structure calculations,[^7^, ^8^, ^9^] using the concepts of ligand, strain and ensemble effects.[^8^, ^10^, ^11^, ^12^, ^13^, ^14^, ^15^]

However, the catalytically most active bimetallic structure will be worthless if it is not stable under operating conditions[^16^, ^17^] because this limits its lifetime and thus prohibits its use in real catalysts. Bimetallic structures at temperatures significantly below their melting temperature typically do not correspond to equilibrium structures,[^18^, ^19^] as exchange and diffusion processes in metals are usually hindered by rather high energetic barriers. On the other hand, the interaction with reactive species can lead to structural changes, in particular at open surface structures.^[20]^

In spite of the technical importance of the thermal stability of bimetallic catalysts, it is fair to say that there are only few theoretical studies addressing this issue.^[21]^ Hence our theoretical understanding of the reactivity of bimetallic surface structures is much more developed than our understanding of the thermal (meta‐)stability of these structures. This is due to the fact that there are successful reactivity concepts relating electronic and structural properties of bimetallic surfaces to their catalytic activity,[^22^, ^23^] but there are no such simple concepts with regard to the stability of bimetallic surfaces. The thermodynamic stability can in principle be derived from density functional theory (DFT) calculations of the corresponding equilibrium structure together with an appropriate consideration of entropy terms to derive the free energy. However, there is no such simple approach with respect to non‐equilibrium stationary structures that are kinetically stabilized by the presence of large barriers hindering the transition to an equilibrium state. Diamond is a well‐known example. Although diamonds are forever, they are only meta‐stable with regard to the more stable graphite structure.

In metals, structural rearrangements typically occur through substitutional changes involving the presence of vacancies.[^24^, ^25^, ^26^, ^27^, ^28^, ^29^, ^30^, ^31^] First, vacancy concentrations can be rather small in metals, and second, the corresponding diffusion barriers are often relatively high. Hence it is often not clear whether metal structures correspond to equilibrium structures, in particular as far as alloys are concerned, because the time scale for equilibration becomes very large because of infrequent diffusion processes. For example, in a study of the stable structures of bimetallic CuPd/Ru(0001) surface alloys, the experimental observed structures were analyzed in Monte Carlo simulations that were performed at a temperature of 600 K close to the annealing temperature used in the preparation of the surface alloy^[18]^ and not at the temperature of 300 K at which the surface alloy structure was recorded by scanning tunneling microscopy (STM) measurements and at which the alloy structure is practically frozen.

However, to model structural changes of bimetallic surfaces requires to perform simulations on macroscopic time scales and mesoscopic length scales. Here the method of choice are kinetic Monte Carlo (kMC) simulations^[32]^ with the rate constants entering this formalism derived via transition state theory^[33]^ from periodic density functional calculations. This approach has already been used extensively to analyse, e. g., structural properties of alloys,[^34^, ^35^] their thermal decomposition^[36]^ or the ordering kinetics in bimetallic nanoparticles.^[37]^ The application of kMC simulations also expands to surface properties concerning growth processes in thin film epitaxy[^38^, ^39^] and adsorption, growth or reaction processes on surfaces.[^40^, ^41^, ^42^] Recently it was shown, that first‐principles kMC (1p‐kMC) methods can be successfully used to elucidate surface chemical processes and heterogeneous catalysis.[^42^, ^43^, ^44^]

In a first step to address the (meta)‐stability of structures in bimetallic systems, we focus here on the vacancy assisted diffusion on single‐atom alloys. These alloys have recently been discussed as efficient catalysts offering the advantage that the active precious metal can be diluted at the atomic limit.^[45]^ In particular, we combine DFT calculations with kMC simulations in order to address the (meta‐)stability of PtRu/Ru(0001), AgPd/Pd(111), PtAu/Au(111) and InCu/Cu(100) single‐atom surface alloys with one additional vacancy per simulation cell. First, the diffusion barriers together with the vibrational frequency in the initial and the transition state have been determined, analyzed and then used to derive the jump rates. These rates are then employed in a kMC formalism in order to simulate the diffusion in the corresponding single‐alloy systems.

## Computational Details

First‐principles electronic structure calculations have been performed using the periodic DFT code Vienna ab initio simulation package (VASP).^[46]^ The electronic cores are described by the projector augmented wave method^[47]^ and exchange‐correlation effects have been taken into account within the generalized gradient approximation (GGA) using a revised version of the Perdew‐Burke‐Ernzerhof (RPBE) functional^[48]^ which has been found to reliably describe properties of bimetallic systems.^[14]^

The metal surface alloys are modelled by periodic slabs consisting of five atomic layers and 6×6 unit cells for Cu(001) and Ru(0001) and 5×5 in the case of Pd(111) and Au(111). The top three layers of the slabs are fully relaxed, while the lowermost two layers are fixed at their bulk positions. A vacuum of 15 Å separates the slabs to avoid any interaction between the periodic images. The wave functions were expanded up to a cutoff energy of 400eV and a [3×3×1] k‐point grid for all surfaces and alloys.

The first‐principles based kinetic Monte Carlo simulations[^32^, ^41^] have been performed employing the general lattice kinetic Monte Carlo (kMC) framework of kmos.^[49]^ For the kMC simulations, the cell with periodic boundary conditions was expanded to 48×48 unit cells. The rates entering the kMC simulation were derived from the DFT calculations using transition state theory (TST).^[50]^

## 2 Results and Discussion

### 2.1 Energetics of the Single‐Atom Surface Alloys

In this work we consider surfaces that consist of one vacancy and one foreign atom per unit cell leading to a single‐atom surface alloy. In order to assess the stability of the studied structures in the single‐atom surface alloys, we first determined formation energies using DFT.^[51]^ The vacancy formation energy is given by

(1) $$\Delta H_{f}^{V}=\left(E_{surf}^{V}+E_{bulk}^{M}\right)-E_{surf}\;,$$

where $E_{surf}^{V}$ is the total energy of the metal or alloy surface with the vacancy per unit cell and $E_{bulk}^{M}$ is the bulk cohesive energy of the metal M that has been occupying the vacancy site before the formation of the vacancy, and *E_surf_* is the energy of the surface without the vacancy. This means that we assume that the metal atom whose extraction has lead to the vacancy is inserted into a metal bulk reservoir which for example could also be realized by attachment to a corresponding kink site of the metal.

The alloy formation energy

(2) $$\Delta H_{f}^{alloy}=E_{alloy}-E_{surf}-\left(E_{bulk}^{M_{a}}-E_{bulk}^{M_{b}}\right)$$

reflects the process that a metal atom M_*a*_ is extracted from the surface whose energy is given by *E_surf_* and is replaced by a foreign metal atom M_*b*_ leading to the formation of the single‐atom surface alloy whose energy is given by *E_alloy_*. Both metal atoms M_*a*_ and M_*b*_ are assumed to be exchanged with the corresponding bulk metal reservoir.

Both the vacancy surface and the alloy formation energy of the considered systems based on a Ru(0001), Pd(111) and Au(111) substrate, respectively, are listed in Table 1. With V@…, the formation of a vacation is denoted, M@… reflects the formation of a single‐atom surface alloy with M as the foreign metal atoms, and V@M@… describes the formation of a vacancy next to the foreign atom of a M@… single‐atom surface alloy.

**Table 1:** Enthalpies of formation (H_f_) in (eV) for introduction of a vacancy (V) and a foreign atom (Pt,Ag) into the pure surfaces.

surface	Δ H_f_ (eV)
V@Ru(0001)	1.46
Pt@Ru(0001)	−0.72
V@Pt@Ru(0001)	1.34
V@Pd(111)	0.80
Ag@Pd(111)	−0.29
V@Ag@Pd(111)	0.72
V@Au(111)	0.62
Pt@Au(111)	0.25
V@Pt@Au(111)	0.74

According to Table 1, the more noble the metal is, the less costly the formation of a surface vacation is, i. e., the vacancy formation energy scales with the cohesive energies of the metals. The vacancy formation energies indicate that the number of vacancies in thermal equilibrium should be rather low. Taking the Boltzmann factor $\exp(-H_{f}/k_{B}T)$ as measure of the vacancy concentration, at room temperature even for Au(111) with the lowest vacancy formation energy a vacancy concentration of below 10^−10^ results.

To form a single‐atom surface alloy with a foreign atom that has a lower cohesive energy than the host is energetically favorable, even if the foreign atom is larger, as the examples Pt@Ru(0001) and Ag@Pd(111) confirm. Note that the lattice mismatch between Ru(0001) and Pt(111) is about 2.5 % and almost 5 % for Ag(111) and Pd(111), so naturally, Pt and Ag exert a strain of their neighbouring atoms. Still, the electronic interaction corresponding to the ligand effect obviously over‐compensates the strain introduced through the geometric effect because of the different atom sizes. In contrast, the replacement of a surface atom by a foreign atom with a higher cohesive energy is energetically unfavorable, as the result for Pt@Au(111) shows which has been obtained before.[^52^, ^53^] Overall, all these results can be explained by the energy gain or cost, respectively, to change a 3D environment to 2D and vice versa, irrespective of the size of the metal atoms. The calculated values of the vacancy and alloy formation energies are consistent with STM results by Behm et al.[^54^, ^55^, ^56^, ^57^] First, they found in general small vacancy concentrations, and second, the availability of monomer, dimer, and trimer ensembles of foreign atoms in AgPd/Pd(111) and PtRu/Ru(0001) are comparable. Still, it should be noted that also energetically unfavorable surface alloys such as PtAu/Au(111) have been observed^[58]^ provided that the annealing temperatures are not too high. This confirms that experimentally observed surface alloys are not necessarily in thermal equilibrium.

Finally, we address the question whether the formation of a vacancy‐foreign metal atom pair adjacent to each other is more favorable than having both separated from each other. This can be derived by comparing the formation energies of V@… with those of V@M@…We find the same trends as for the alloy formation energies. For Pt@Ru(0001) and Ag@Pd(111), it is energetically more favorable to have the vacancy and the foreign atom adjacent to each other because then one bond with the less strongly interacting foreign atom is broken whereas it is the other way around for Pt@Au(111). Hence thermodynamically it not preferred that in PtAu/Au(111) a Pt atom and a vacancy form a pair.

**Figure 1:**
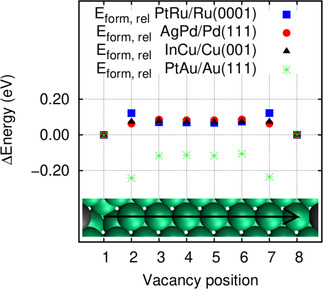
Change in the vacancy formation energy as a function of the distance between vacancy and foreign atom with respect to the vacancy and the foreign atom being adjacent to each other (position 1 and 8) calculated using a 9×9 unit cell.

In Figure 1, we have analyzed the interaction between a foreign atom and a vacancy in more detail by plotting the change in the vacancy formation energy as a function of the distance between the vacancy and the foreign atom using a 9×9 unit cells. Positions 1 and 8 with the foreign atom and the vacancy adjacent to each other act as a reference. In Figure 1, we have also included the system InCu/Cu(001) which will be discussed below. For all considered systems, the formation energy does hardly change any more when the vacancy and the foreign atom are three and more lattice sites apart from each other. The values for the larger distance roughly correspond to the formation energies for V@… given in Table 1, the slight discrepancies are due to the different size of the surface unit cell. Interestingly, both the systems PtRu/Ru(0001) and PtAu/Au(111) show the strongest change when vacancy and foreign atom are second nearest neighbours.

For both systems, in these cases the ligand effect becomes even stronger. In PtRu/Ru(0001), at a second nearest neighbour distance a vacancy is formed with only Ru−Ru bonds being broken, one of which is even stronger as one Ru atom is located adjacent to the foreign Pt atom which does not interact so strongly with the Ru atom. Conversely, in PtAu/Au(111) at a second nearest neighbour distance, the vacancy formation is only associated with the breaking of weak Au−Au bonds, one of which is even weaker as one of the Au atoms is more strongly bound to the adjacent Pt atom. This also means that for PtAu/Au(111), the second nearest neighbour site is the energetically most preferred site. So although the vacancy does not prefer to be located adjacent to the foreign atom, it is still energetically favorable to stay close to the foreign atom.

Finally we like to note that the incorporation of a vacancy in subsurface layers and further into the bulk is energetically less favorable than a surface vacancy^[59]^ because a larger number of bonds needs to be broken. Hence only surface vacancies are considered in this study.

**Figure 2:**
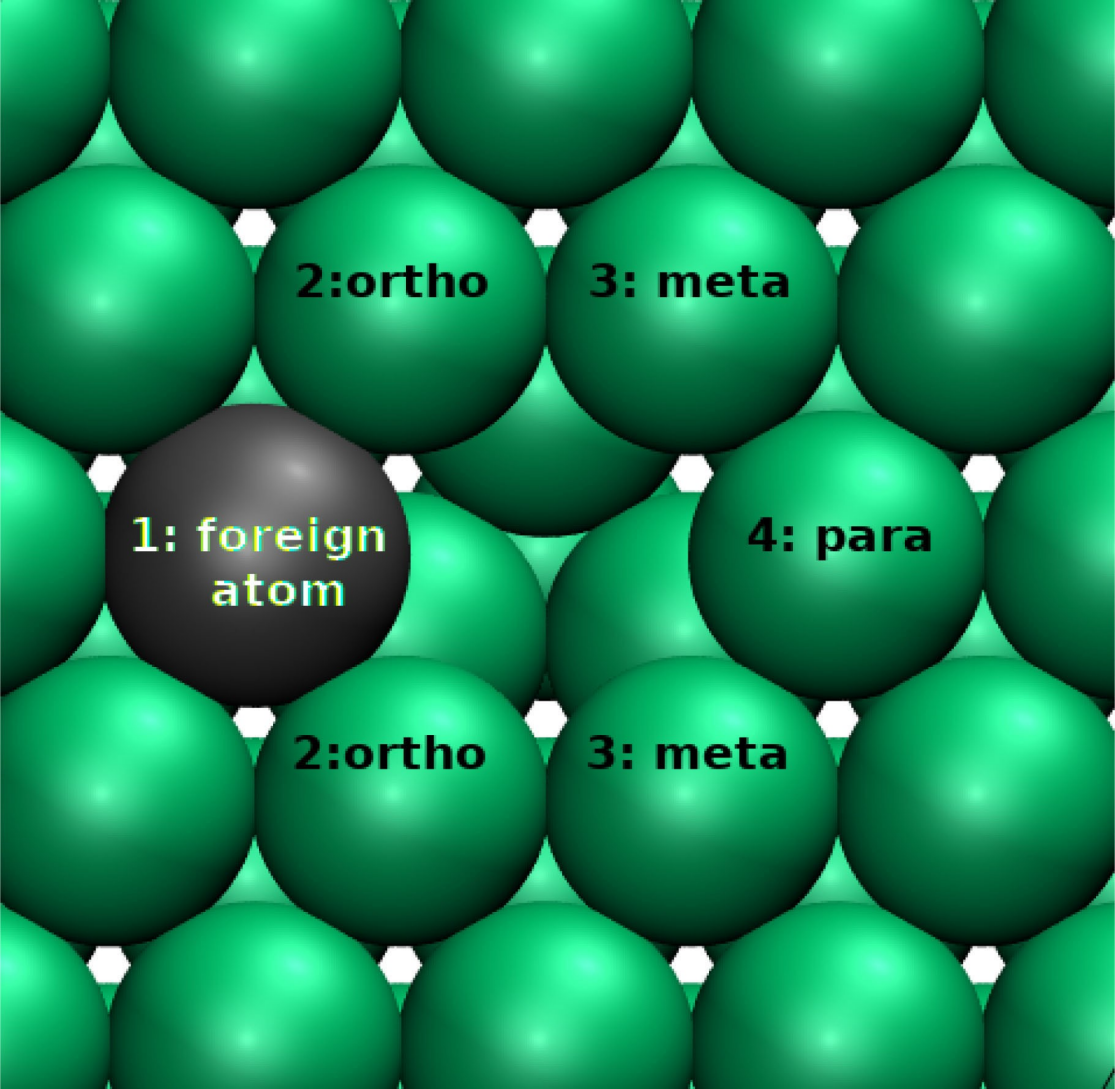
Nomenclature of the atoms that can move via vacancy‐assisted diffusion on a hexagonal surface when a single foreign atom is present at position 1 adjacent to the vacancy.

**Figure 3:**
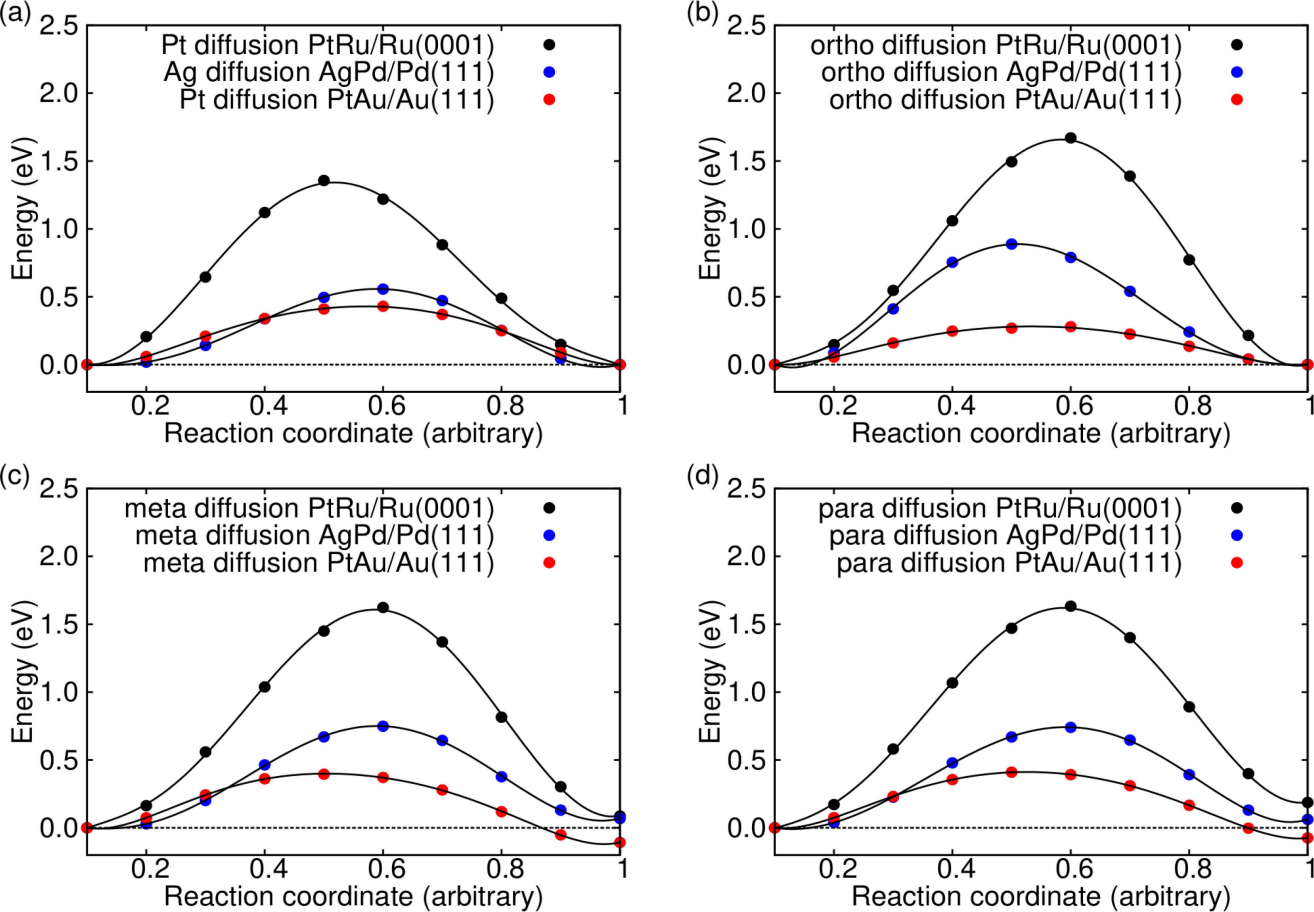
Minimum energy paths for the diffusion of the foreign atom (a) and *ortho* (b), *meta* (c) and *para* (d) diffusion in the PtRu/Ru(0001) (black symbols), AgPd/Pd(111) (blue symbols) PtAu/Au(111) (red) surfaces determined by NEB calculation.

Changes in the configuration of a surface alloy can only occur via vacancy diffusion as long as no diffusion *on* the surface is possible. Hence the vacancy acts as a “mixer”. In a pure single‐metal surface, vacancy diffusion can be regarded as a random walk with every possible vacancy diffusion step being equally probable. However, the presence of a foreign atom breaks this symmetry, as illustrated for a (111) surface in Figure 2. In total, four symmetrically inequivalent diffusion events can occur, either the foreign atom can make a jump or the substrate atoms at the *ortho*, *meta* and *para* positions relative to the location of the foreign atom. For some of the events, initial and final positions are also inequivalent so that the barriers for the forward and backward jumps become different. The corresponding diffusion barriers are all collected in Table 2 and compared with the vacancy diffusion barriers for the pure surfaces. Furthermore, for the systems PtRu/Ru(0001) and AgPd/Pd(111) the NEB energetics along the minimum energy diffusion paths for the foreign atoms and for the *ortho*, *meta* and *para* diffusion are plotted in Figure 3.

**Table 2:** Surface diffusion barriers (E_b_) in eV for the diffusion of the foreign atom and of the substrate atoms initially in the ortho, meta and para positions, as illustrated in Figure 2. In addition, the vacancy diffusion barrier for the pure surfaces are included. Barriers for the forward and backward diffusion are given. If only one number is given, then both barriers are equal.

	E_b_ (eV)		
	PtRu/Ru(0001)	AgPd/Pd(111)	PtAu/Au(111)
		forward/backward	
foreign atom	1.36	0.56	0.81
*ortho*	1.67	0.89	0.28
*meta*	1.62/1.49	0.74/0.67	0.40/0.50
*para*	1.63/1.45	0.74/0.68	0.41/0.48
pure surface	1.55	0.75	0.43

It is important to recall that the primary influence on mobility is the number and strength of broken bonds and the size of the atom which is moved.^[24]^ In general, diffusion becomes more hindered with increasing size of the atom and for a stronger interaction with the other atoms. A larger radius induces strain on the nearest neighbours, while an increased interaction leads to stronger bonds. In the case of PtRu/Ru(0001) and AgPd/Pd(111), the foreign atom is larger, but also less reactive, which means that the two effects are competing with each other. However, as Table 2 shows, similar as in the case of the surface alloy formation energies, in these two systems the electronic ligand effect obviously overcompensates the geometric strain effect as the diffusion barriers for the foreign metal atoms are smaller than the self‐diffusion barriers of the host metal. In principle, the dominance of the ligand effect is also true for the PtAu/Au(111) system, however, here the ligand effect leads to a larger diffusion barrier for the more reactive but smaller Pt atom in a matrix of less strongly interacting, but larger Au atoms compared to the self‐diffusion barriers of the Au atoms.

With respect to the self‐diffusion barriers we find for PtRu/Ru(0001) and AgPd/Pd(111) that *meta* and *para* diffusion is hindered by smaller barriers than the *ortho* diffusion. Apparently both the ligand and the strain effect make the transition state even more unfavorable than the initial state. Both the diffusion from the meta and the para position exhibit lower barriers for the backward diffusion which simply reflects the fact that in these systems the vacancy adjacent to the foreign atom is more favorable than the vacancy at a second‐nearest neighbour distance to the foreign atom (see Figure 1). Thus the barriers for the backward diffusion are also smaller than the vacancy diffusion barrier on the pure surface.

Again, the PtAu/Au(111) surface shows an opposite behavior because here the foreign atom is smaller, but more strongly interacting than the host metal atoms. Therefore now *ortho* diffusion is preferred compared to *meta* and *para* diffusion, and in the latter two cases the backward diffusion becomes more hindered than the forward diffusion. Still, as in the PtRu/Ru(0001) and AgPd/Pd(111) systems these results can only be understood under the assumption that the ligand effect overcompensates the geometric effect.

### 2.2 Diffusion in Single‐Atom Surface Alloys

The main goal of this study is to address the (meta‐)stability of bimetallic surface structures. In the previous section, we have studied the energetics of single‐atom surface alloys together with a vacancy using periodic density functional theory calculations. Now we will consider the question whether the considered structures are stationary based on kinetic Monte Carlo simulations. The transition rates entering the kMC formalism will be obtained using Arrhenius expressions derived from the DFT calculations based on transition state theory.^[50]^ The prefactor *ν*_0_ entering this expression that can be interpreted as an attempt frequency is often approximated to be 10^13^ $\frac{1}{s}$ in order to avoid the considerable numerical effort to derive vibrational frequency. Furthermore, whereas activation energies enter the transition rates *k_j_* exponentially, the frequencies only enter linearly so that they have a minor influence on the rates. Still, we have derived the prefactors from vibrational frequency calculations within harmonic approximation according to

(3) $$\nu_{0}=\frac{1}{2\pi }\frac{\prod_{i=0}^{N}\omega_{i}^{\left(0\right)}}{\prod_{i=1}^{N}\omega_{i}^{\left(TS\right)}}$$

These vibrational frequencies have to be determined both at the ground state ($\omega_{i}^{\left(0\right)}$ ) and at the TS ($\omega_{i}^{\left(TS\right)}$ ). In order to keep the computational effort in a manageable range in the finite difference scheme to derive the vibrational frequencies, the unit cell size was reduced to 4×4. Furthermore, the structures of the ground state and TS taken from the NEB calculations have been fixed and finite differences were only taken with respect to the particular atom of interest. The calculated prefactors *ν*_0_ are collected in Table 3. Interestingly enough, the calculated values are all pretty close to the canonical value of 10^13^ $\frac{1}{s}$ which hence would have been a reasonable approximation in this case.

**Table 3:** Calculated results for the pre‐exponential factor ν_0_. Ortho, meta and para refer to the diffusion around the vacancy relative to the foreign atom.

$\nu \left(\frac{1}{s}\right)$			

diffusion	PtRu/Ru(0001)	AgPd/Pd(111)	PtAu/Au(111)
foreign atom	1.06$\cdot 10^{13}$	8.79$\cdot 10^{12}$	1.31$\cdot 10^{13}$
*ortho*	1.67$\cdot 10^{13}$	7.75$\cdot 10^{12}$	8.80$\cdot 10^{12}$
*meta*	1.62$\cdot 10^{13}$	8.42$\cdot 10^{12}$	8.53$\cdot 10^{12}$
*para*	1.31$\cdot 10^{13}$	2.74$\cdot 10^{12}$	9.23$\cdot 10^{12}$
pure surface	9.77$\cdot 10^{12}$	1.09$\cdot 10^{13}$	7.86$\cdot 10^{12}$

The simplest diffusion process is the one of the diffusion of a single vacancy in an otherwise homogeneous metal surface. Then all diffusion events are equivalent with the same rate so that they correspond to a random walk. For such a random walk, the self‐diffusion coefficient D_*s*_ is related to the jump rate *k_j_* according to

(4) $$\begin{array}{c} D_{s}=\frac{k_{j}\cdot a^{2}}{b}. \end{array}$$

For such a self‐diffusion, the mean square displacement is a linear function of the time,

(5) $$\begin{array}{c} <R^{2}\left(t\right)>=\;t\cdot k_{j}\cdot a^{2}=b\cdot D_{s}\cdot t. \end{array}$$

The factor *b* is surface dependent and represents the number of possibilities for diffusion, which is six for hexagonal surfaces and four in the case of square surfaces. Note that this linear dependence of the mean square displacement is only observed when an average over many trajectories *R*(*t*) is done.

Using Eq. 4, we derived the self‐diffusion coefficient (D${}_{s}^{298}$ ) for the diffusion of an isolated vacancy at 298K in Ru(0001), Pd(111) and Au(111) (see Table 4). Even for the soft metal Au, the diffusion coefficient is smaller than 10${}^{-13}\frac{m^{2}}{s}$ . In order to put this diffusion constant into perspective, we like to note that typical diffusion coefficients in liquids at 298K are in the range of 10${}^{-9}\frac{m^{2}}{s}$ .^[60]^ Concerning the metal vacancy diffusion coefficients in Ru(0001), we like to stress another point here. Taking the value of $D_{s}=8.28\cdot 10^{-34}\frac{m^{2}}{s}$ , one can estimate that it takes about $5\times 10^{17}$ seconds for a vacancy in Ru(0001) to move about 100nm at room temperature. Note that the age of the universe is about 5×10^17^ seconds which means that it takes the vacancy about $\frac{1}{100}$ of the age of the universe to move this rather small distance. This illustrates how immobile vacancies are in metal surfaces at room temperature.

**Table 4:** Self‐diffusion coefficients of a surface vacancy in $\frac{m^{2}}{s}$ for the Ru(0001), Pd(111) and Au(111) surfaces at 298K, calculated via a linear fit to the mean square displacement (MSD) and Eq. 4.

$D\frac{298}{s}\left(\frac{m^{2}}{s}\right)$		

surface	MSD	Eq. 4
Ru(0001)	6.89 ⋅ 10^−34^	6.78 ⋅ 10^−34^
Pd(111)	7.06 ⋅ 10^−20^	6.80 ⋅ 10^−20^
Au(111)	1.38 ⋅ 10^−14^	1.23 ⋅ 10^−14^

**Figure 4:**
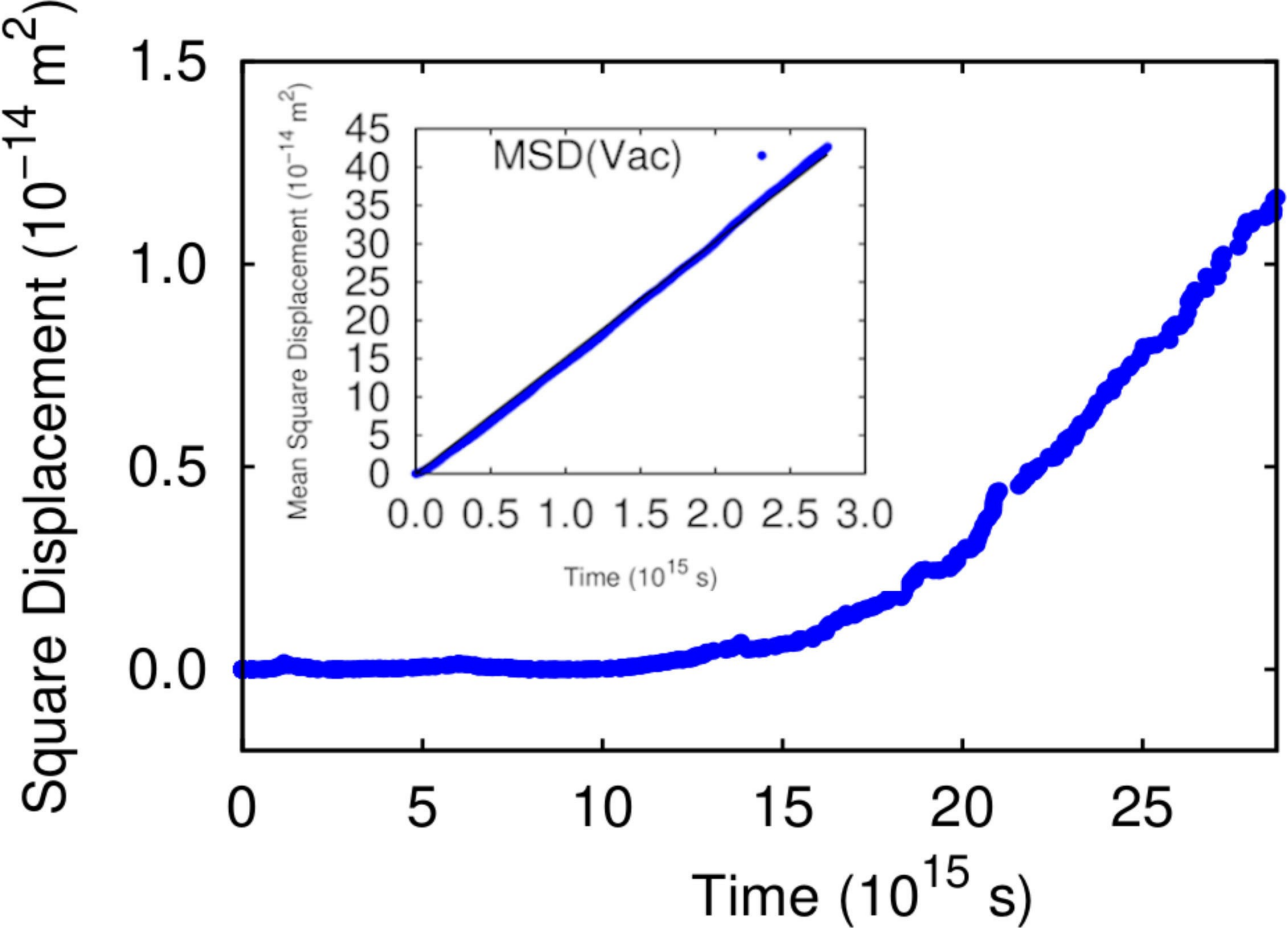
Square displacement of a vacancy in Ru(0001) in the presence of a Pt atom at 298 K along one kMC trajectory. Inset: Mean square displacement of an isolated vacancy on Ru(0001) at 298 K averaged over 10,000 kMC trajectories.

Instead of deriving the self‐diffusion constant *D* directly from the jump rates according to Eq. 4, it is also possible to evaluate *D* from the mean‐square displacement using Eq. 5. We have run 100 different kMC trajectories, and for each trajectory we have taken 100 different initial points equally spaced in time. This means that the mean‐square displacement has been derived by sampling of 10,000 trajectories. As an example, the results for a vacancy in Ru(0001) are plotted in the inset of Figure 4. As can be seen from Table 4, the self‐diffusion coefficients calculated from the kMC simulation and by Eq. 4 are in the same range but differ by up to more than 10 %. This shows that even for such a seemingly simple process like a random walk on a hexagonal lattice an extensive sampling is required to derive a reliable diffusion coefficients from the mean‐square displacement as a function of time.

We now turn to the diffusion of one vacancy and one foreign atom within the 48×48 unit cell of the kMC simulations which means that we assume a constant vacancy concentration of 0.04 % and a 1 : 1 ratio between foreign atom and vacancy. In Figure 4, the square displacement of the vacancy in the PtRu/Ru(0001) single‐atom alloy surface at room temperature along one kMC run is plotted illustrating the typical events that occur in these systems. For the first $1.5\times 10^{14}\;\;$ s, the vacancy hardly moves away from the initial position. This is due to the attraction between the vacancy and the Pt atom so that the vacancy basically always returns to the Pt atom leading to almost no net displacement. After $1.5\times 10^{14}$ s, the vacancy then separates from the Pt atom and does not return back. Hence a random walk of the single surface vacancy starts, as described above, exhibiting a linear dependence of the square displacement on time.

**Figure 5:**
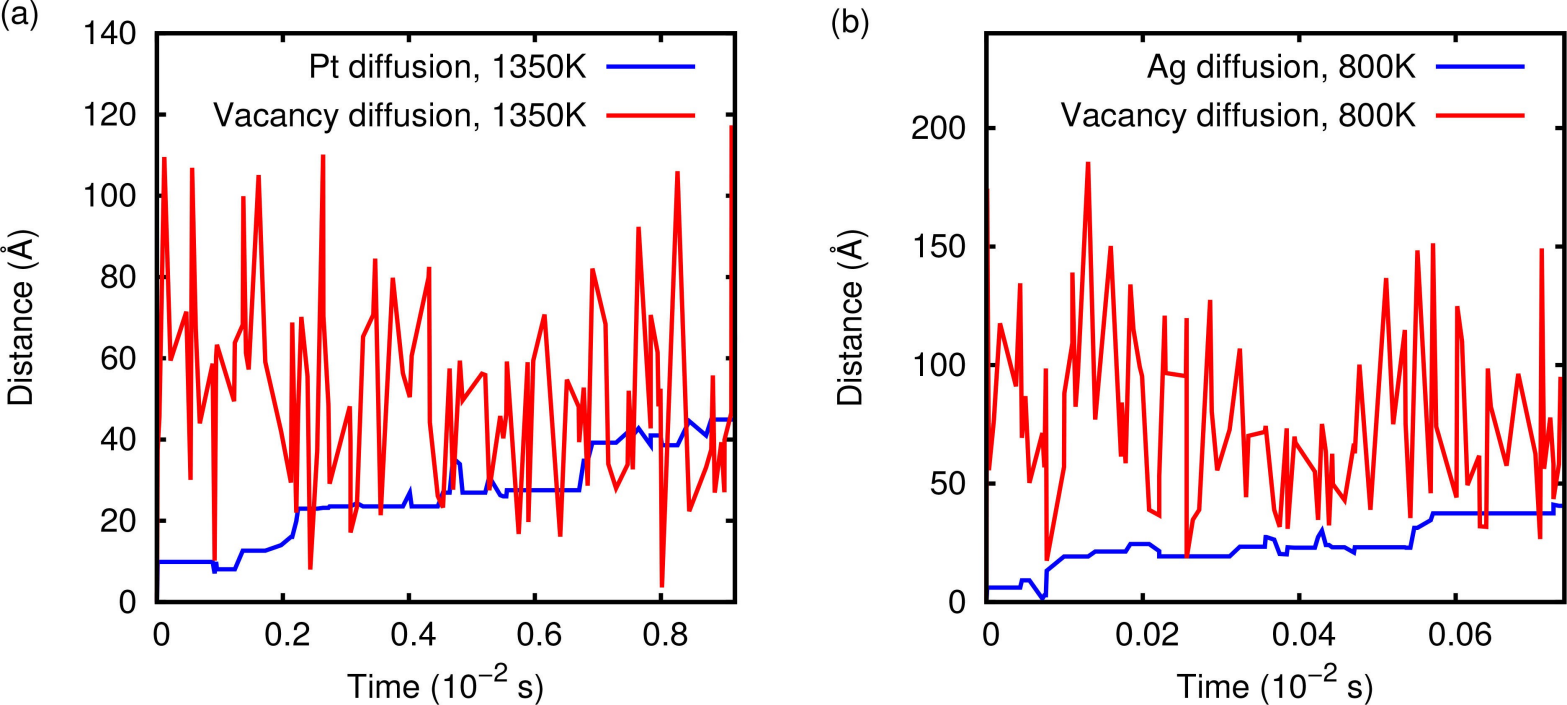
Distance of the foreign atom and the vacancy as a function of time for in total 10^6^ kMC steps for the PtRu/Ru(0001) system at 1350 K (a) and for the AgPd(111) system at 800 K (b).

In Figure 5 we have plotted the distance of the foreign atom and the vacancy for in total 10^6^ kMC steps for PtRu/Ru(0001) at 1350K and for AgPd(111) at 800 K. Due to the different absolute values of the jump rates in these two systems and at two temperatures (see Table 5), the 10^6^ kMC steps correspond to two different time scales that differ by about one order of magnitude. The foreign atoms experience long periods of immobility due to the absence of the vacancy at a neighboring site. It is important to realize that at higher temperatures the relative difference between different rates become smaller. Table 5 compares the jump rates for the foreign atoms adjacent to the vacancy and for the isolated vacancy in the pure metal for the three systems PtRu/Ru(0001), AgPd/Pd(111) and PtAu/Au(111) at four different temperatures. For example, whereas the rates of the two listed jump events differ at 298 K by more than two orders of magnitude in the system PtRu/Ru(0001), they only differ by one order of magnitude at 1350 K. For AgPd/Pd(111) at 800 K, in contrast, the jump rate of the foreign atom is larger than the vacancy jump rate by a factor of 15. This means that the vacancy jumps less frequently for AgPd/Pd(111) at 800 K than for PtRu/Ru(0001) at 1350 K per kMC steps which results in a lower distance of the vacancy from the initial position after the same number of kMC steps. As the propagation of the foreign atom is directly linked to the mobility of the single vacancy, also the Ag atom stays closer to the initial position than the Pt atom.

**Table 5:** Dependence of the jump rates k in $\frac{1}{s}$ of Pt in the Ru(0001) surface, Pt in the Au(111) surface and Ag in the Pd(111) surface on the temperature T in K. The rates k_Ru_, k_Pd_ and k_Au_ denote the diffusion of the isolated vacancy in the pure metals.

	T(K)			
k $\left(\frac{1}{s}\right)$	298	400	800	1350
k${}_{\mathrm{Pt}}^{\mathrm{Ru}(0001)}$	8.35 ⋅ 10^−11^	1.42 ⋅ 10^−4^	5.01 ⋅ 10^4^	1.52 ⋅ 10^8^
k_Ru_	5.46 ⋅ 10^−14^	2.71 ⋅ 10^−7^	1.63 ⋅ 10^3^	1.57 ⋅ 10^7^
k${}_{\mathrm{Ag}}^{\mathrm{Pd}(111)}$	2.37 ⋅ 10^3^	8.52 ⋅ 10^5^	2.74 ⋅ 10^9^	7.34 ⋅ 10^10^
k_Pd_	1.46	3.93 ⋅ 10^3^	1.98 ⋅ 10^8^	1.63 ⋅ 10^10^
k${}_{\mathrm{Pt}}^{\mathrm{Au}(111)}$	0.11	5.72 ⋅ 10^2^	7.57 ⋅ 10^7^	9.23 ⋅ 10^9^
k_Au_	4.75 ⋅ 10^5^	4.30 ⋅ 10^7^	2.07 ⋅ 10^10^	2.57 ⋅ 10^11^

Of course, with respect to the absolute time scale also the absolute values of the jump rates matter. For example, the lower the jump rate is, the higher the annealing temperature has to be in order to get an equilibrium distribution. The differences in the absolute jump rates well reflect the annealing temperatures experimentally used for these systems, namely 1350K for PtRu/Ru(0001),^[54]^ 800 K for AgPd/Pd(111,^[56]^ and 900 K for PtAu/Au(111).^[61]^

**Figure 6:**
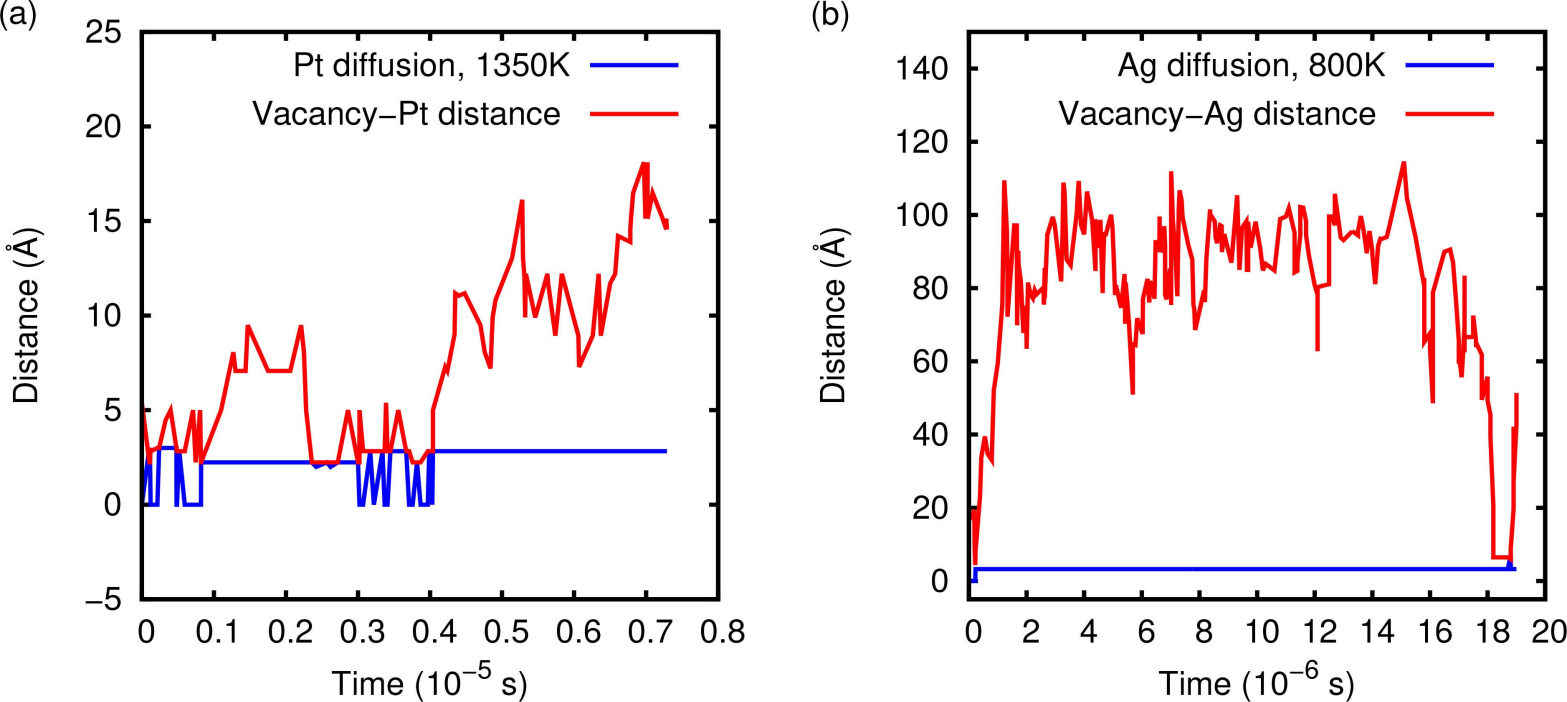
Distance of the foreign atom and the vacancy as a function of time for the PtRu/Ru(0001) system at 1350 K (a) and for the AgPd/Pd(111) system at 800 K (b) for the initial steps up to a simulation time of about 10^−5^ s and 10^−6^ s, respectively.

Still, in Figure 5 the exact correlation between the propagation of the vacancy and the foreign atom is not directly visible due to the low time resolution which does not allow to capture every single step. Therefore we increased the time resolution of the kMC simulation by only plotting the initial steps up to about a simulation time roughly corresponding to 10^−6^ s in Figure 6. Indeed it becomes obvious that the propagation of the foreign atom is only possible when the vacancy is at a neighboring site. Furthermore, it demonstrates how far the vacancy and the foreign atom separate from each other before recombining again which is quite different for the considered systems depending on the height of the diffusion barriers.

Regarding the PtRu/Ru(0001) simulation, it is also interesting to note that many of the Pt migration events corresponds just to jumps back and forth. This is due to the fact that the barrier for the movement of the foreign atom is considerably smaller than the barriers for the *ortho*, *meta*, and *para* processes (see Table 2). In contrast, for the PtAu/Au(111) system the surface diffusion barrier for the foreign atom is much larger than all other barriers involving the vacancy. Consequently, the foreign atom and the vacancy most often rather quickly separate from each other leaving the foreign Pt atom immobile, as confirmed by our kMC simulations.

Thus the diffusion of the foreign atom is coupled to the diffusion of the vacancy. In such a situation, the diffusion coefficient can be derived^[26]^ according to:

(6) $$\begin{array}{c} D_{x}=a^{2}k_{x}f_{x}\cdot \exp\left(-\frac{\Delta H_{f}^{vac}\left(surf\right)+E_{binding}^{vac-x}}{k_{B}T}\right) \end{array}$$

Here, *a* corresponds to the lattice parameter, k_*x*_ is the jump rate proportional to $\exp(-E_{b}/k_{B}T)$ , ΔH${}_{f}^{vac}$ the enthalpy of forming a vacancy in the clean surface, E${}_{binding}^{vac-x}$ the binding energy of the vacancy and the foreign atom estimated by the energy difference of having a vacancy as nearest neighbour and second nearest neighbour, and f_*x*_ is a foreign atom correlation factor.[^62^, ^63^, ^64^] The exponential factor couples the diffusion barrier with the probability that a vacancy is present adjacent to the foreign atom.^[28]^

**Figure 7:**
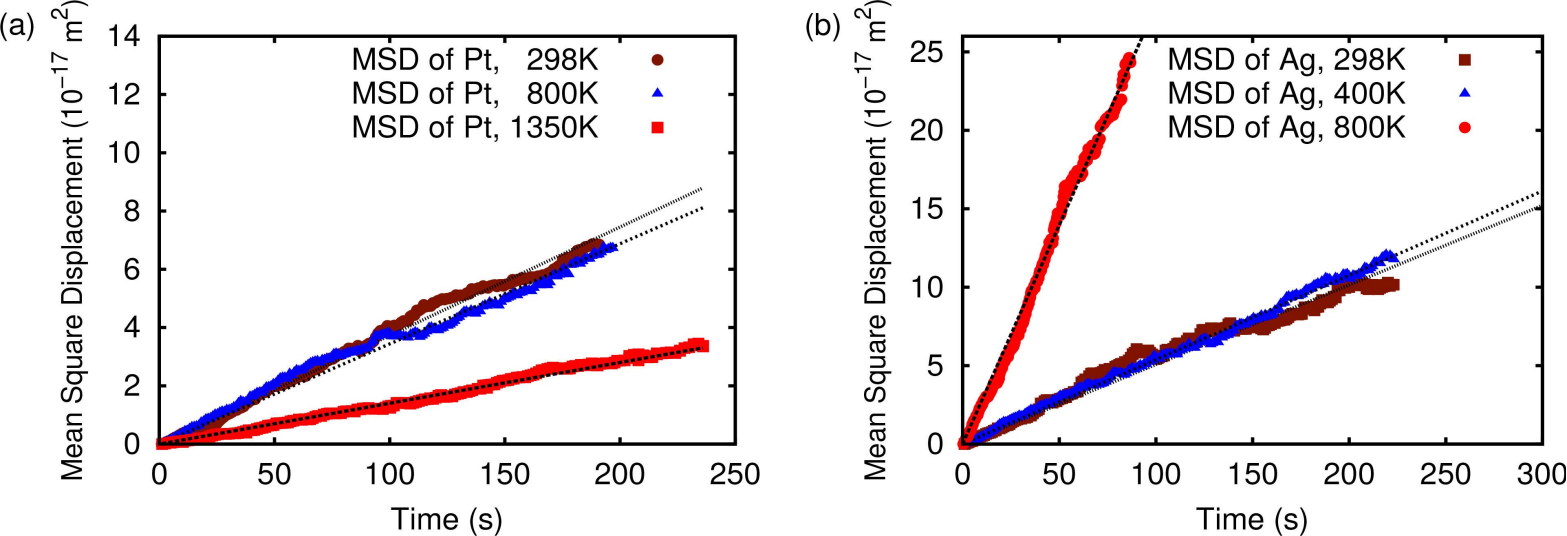
Mean square displacement for Ag in Pd(111) (a) and Pt in Ru(0001) (b) in the presence of a vacancy at different temperatures. For the time‐axis following scaling divisors have been applied: < R^2^(t)> (Pt) at 1350K: 6 ⋅ 10^−5^, <R^2^(t)> (Pt) at 298K: 5 ⋅ 10^16^, <R^2^(t)> (Ag) at 800 K: 6 ⋅ 10^−5^, <R^2^(t)> (Ag) at 400 K: 0.9, <R^2^(t)> (Ag) at 298 K: 2 ⋅ 10^3^.

Since the diffusion of foreign atoms is dependent on the return probability of the tracer in addition to the temperature and energy barriers, deriving the diffusion coefficient via the kMC simulation is more complicated than for the vacancy diffusion. In order to reproduce the linear relation between the mean square displacement <R^2^(Ag, Pt)> and time *t*, we enlarge the sampling time Δ *t* to a time range in which the foreign atom is moving. In this way, the period of time in which the vacancy is separated from the foreign atom so that it can not move can be bypassed. These periods are the flat regions in the plot of the distance as a function of time in Figure 5. The resulting mean square displacement together with the linear fits are presented in Figure 7, and the corresponding diffusion coefficients of the foreign atoms calculated using Eq. 6 and derived from a linear fit to the mean square displacements plotted in Figure 7 are compared in Table 6. Note, that due to the considerable differences in Δ *t* for the different considered systems, the time‐axes in Figure 7 were divided by scaling factors given in the captions of Figure 7 to normalise Δ t to 1. In this way all regressions can be plotted in one figure.

**Table 6:** Diffusion coefficients of foreign atoms (D_x_) in $\frac{m^{2}}{s}$ for the Pt in the Ru(0001), Ag in the Pd(111) and Pt in the Au(111) surface at different temperatures calculated from the mean‐square displacement (Eq. 5) and from Eq. 6.

	$D_{x}\left(\frac{m^{2}}{s}\right)$		
surface	temperature	MSD	Eq. 6
	298 K	1.22 ⋅ 10^−39^	8.43 ⋅ 10^−55^
PtRu/Ru(0001)	800 K	5.84 ⋅ 10^−20^	6.10 ⋅ 10^−24^
	1350 K	4.09 ⋅ 10^−16^	8.15 ⋅ 10^−17^
	1500 K	1.33 ⋅ 10^−15^	2.60 ⋅ 10^−15^
	2000 K	2.16 ⋅ 10^−14^	4.92 ⋅ 10^−13^
	298 K	6.31 ⋅ 10^−23^	4.28 ⋅ 10^−31^
AgPd/Pd(111)	400 K	1.30 ⋅ 10^−19^	1.59 ⋅ 10^−24^
	800 K	7.82 ⋅ 10^−15^	1.51 ⋅ 10^−15^
	1350 K	4.53 ⋅ 10^−13^	6.55 ⋅ 10^−12^
	1500 K	7.22 ⋅ 10^−13^	2.21 ⋅ 10^−11^
	298 K	0	0
PtAu/Au(111)	400 K	0	0
	900 K	0	0

Here we now find significant deviations between the diffusion coefficients derived from the mean‐square displacement and those obtained using Eq. 6, in particular at low temperatures. As Figure 6a shows, the vacancy and the foreign atom can exhibit a rather concerted motion. As explained in the discussion of Figure 6, the foreign atom often just jumps back and forth because of the differences in the migration barriers for the movement of the foreign atom compared to the barriers for the *ortho*, *meta*, and *para* processes. Such correlations are obviously not fully reflected in Eq. 6. At high temperatures when the relative difference between the different rates become less pronounced, these correlations matter less so that Eq. 6 better captures the coupled diffusion of the foreign atom and the vacancy.

**Figure 8:**
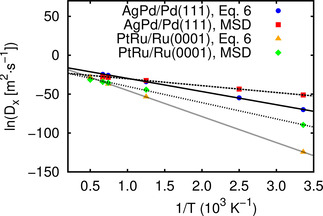
Logarithm of the diffusion coefficients of foreign atoms (D_x_) for Ag in Pd(111) and Pt in Ru(0001) plotted as a function of the inverse temperature derived from Eq. 6 and from the mean‐square displacement (MSD, Eq. 5) along the kMC trajectories. The slope of the kMC results corresponds to effective activation barriers of 0.76eV for Ag in Pd(111) and 1.80 eV for Pt in Ru(0001), respectively.

In order to understand the differences between the diffusion coefficients derived from Eq. 6 and from the mean‐square displacement (MSD, Eq. 5) along the kMC trajectories, we have plotted their logarithm as a function of the inverse temperature in Figure 8. First of all it is obvious that also the diffusion coefficients derived from the mean‐square displacement exhibit a nice linear behavior in this Arrhenius plot indicating that the same effective activation barrier is operative in the whole temperature range covered in the kMC simulations. Second, for both systems the slope for the diffusion coefficients derived from the mean square displacement is smaller than for the diffusion coefficients calculated according to Eq. 6. This means that the effective activation barrier is smaller than the expression entering the exponential of Eq. 6. As mentioned above, one could interpret the term $\exp\left(-\Delta H_{f}^{vac}\left(surf\right)/k_{B}T\right)$ in Eq. 6 as being related to the probability to create a vacancy adjacent to the foreign atom. However, in the systems considered in this study, the vacancy does not need to be created but is already present on the surface, as is also the foreign atom. Hence it should rather give the probability that an existing vacancy is adjacent to the foreign atom which is obviously underestimated by the exponential expression given above.

The slope of the kMC results in Figure 8 corresponds to effective activation barriers of 0.76 eV for Ag in Pd(111) and 1.80 eV for Pt in Ru(0001), respectively, which are higher than the diffusion barriers of the foreign atom listed in Table 2, by 0.20 eV and 0.44 eV, respectively, for the two considered systems. Hence the apparent activation barriers are indeed increased with respect to the simple exchange mechanism between vacancy and foreign atom, but only to a value in the range of the other diffusion barriers that contribute to the movement of the foreign atom (see Table 2).

Due to the immobility of Pt in Au(111) found in the kMC simulations, as discussed above, we can only assign D${}_{Pt}=0$ to the diffusion coefficient from the mean‐square displacement of Pt in Au(111) which in fact agrees with the result derived from Eq. 6. Indeed, the time Δ *t* between to vacancy diffusion events for the vacancy is in the range of $10^{-14}-10^{-12}$ s, whereas a successful Pt diffusion event can take 10^14^ s which makes it practically impossible to observe such an event in the kMC simulations.

### 2.3 Multi‐Lattice‐Spacing Jumps of InCu/Cu(001)

In addition to the three transition metal alloys, the square InCu/Cu(001) surface alloy was investigated, motivated by a scanning tunneling microscopy study of Frenken et al.[^27^, ^28^] They observed multi‐lattice‐spacing jumps of the In atom at room temperature within a time period of 20 seconds, separated by long time intervals of immobile In atoms in the order of two minutes. Based on diffusion barriers computed by the embedded‐atom method (EAM) and the enumeration of possible trajectories,^[29]^ they proposed a mechanism in which the In atoms stay immobile until a single vacancy mediates the diffusion process. The multi‐lattice‐spacing jumps then occur via successive exchanges with a surface vacancy that could not to be monitored in the experiment due to the low temporal resolution of the STM imaging.[^27^, ^28^]

**Figure 9:**
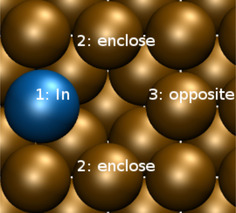
Nomenclature of the atoms that can move via vacancy‐assisted diffusion on the the square InCu/Cu(001) surface with the In atom at position 1.

Due to the square symmetry of the Cu(001) surface, the number of possible diffusion processes for the In atom and the vacancy being adjacent is reduced in comparison to a hexagonal surface. These possible processes are illustrated in Figure 9. Table 7 compares the corresponding calculated vacancy diffusion barriers determined using DFT with those derived with the EAM method by Frenken et al.^[29]^ First of all, our calculations confirm that the barriers are low enough to enable vacancy diffusion in InCu/Cu(001) at room temperate. Still, there are qualitative and quantitative differences between DFT and EAM results. DFT predicts all diffusion barriers to be smaller than EAM which is in particular true for exchange of the vacancy and the In atom. Furthermore, EAM predicts the vacancy jumps to enclose and opposite positions to be hindered by smaller barriers than the vacancy jumps on a pure Cu(001) surface, whereas DFT yields that all the barriers are rather similar. However, both DFT and EAM qualitatively agree that the presence of the In atom increases the *enclose* diffusion barrier relative to the *opposite* diffusion barrier, which is consistent with the difference between *ortho* and *para* processes in the case of PtRu/Ru(0001), and AgPd/Pd(111) surface alloy, which results when a larger foreign atom is embedded in a metal host with smaller atom size.

**Table 7:** Vacancy diffusion barriers E_b_ in eV on InCu/Cu(001) with the nomenclature described in Figure 9, determined using DFT‐RPBE calculations and the EAM method.^[29]^

diffusion	E_b_(RPBE)	E_b_(EAM)
	forward	backward
In	0.06/–	0.24/–
enclose	0.40/0.39	0.50/0.67
opposite	0.36/0.24	0.38/0.53
pure Cu(001)	0.39/–	0.59/–

Using the calculated barriers transformed to migration rate via transition‐state theory, we performed kMC simulations with one vacancy and one In atom present on Cu(001) in a periodic setup with an 47×47 unit cell. Note that this corresponds to a vacancy concentration of about 4$\cdot 10^{-4}$ which is significantly larger than the concentration of about 10^−9^ present in the experiment at room temperature.^[28]^ This basically means that we will not be able to reproduce the long time of inactivity of the In atom, but we should be able to address the multi‐lattice‐spacing jumps.

**Figure 10:**
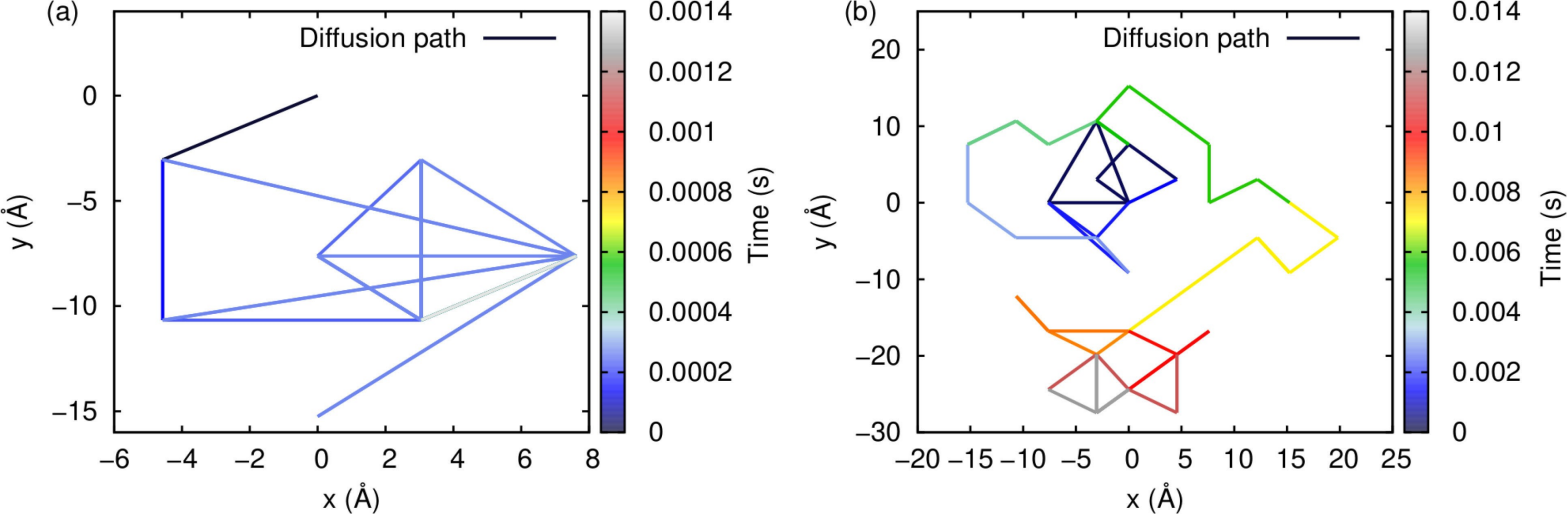
The diffusion path of the In atom in Å at RT at different time resolutions. a) 100 images with a total stepnumber of 10^6^ steps and a simulation time of 1.38$\cdot 10^{-3}$ s and b) 100 images with total stepnumber of 10^7^ steps and a simulation time of 1.27$\cdot 10^{-2}$ s. The colour code represents the time evolution along the diffusion path of the In atom.

Due to the fact that the barrier for In atom jumps in the presence of the vacancy is so low, most of the kMC steps are associated with the flipping of the vacancy and the In atom. A true migration of the In atom can only occur through a combination of Cu jumps from the enclose and the opposite sites with a subsequent recombination of the In atom with the vacancy. In order to see whether our setup is able to reproduce the multi‐lattice‐spacing jumps observed in the experiment, in Figure 10 we have plotted trajectories of the In atoms corresponding to 100 images with different step resolutions of 10^4^ and 10^5^, corresponding to 1.38$\cdot 10^{-5}$ s and 1.27$\cdot 10^{-4}$ s time resolution, respectively. This means that not necessarily all positions of the In atom along the trajectory are recorded, as can well happen in a STM experiment. The time evolution of the steps along the trajectory is colour‐coded from blue to red. For example, the colour‐coding in Figure 10a demonstrates that In jumps only occurred during the first 0.0004 s of the trajectory, in the remaining 0.001s the In atom did not move any further.

Figure 10a demonstrates that within 10^6^ steps or 1.38$\cdot 10^{-3}$ s the In atoms can propagate about 10^−15^ Å. Note that after about 4$\cdot 10^{-4}$ s, the In atom becomes immobile because the vacancy has been separated from the In atom. When we coarse‐grain the resolution by a factor of 10 (Figure 10b), then we obtain a rather continuous propagation of the In atom, indicating that the vacancy after separation always returns to the In atom within 1–10 ms. The total distances from the initial point within 10ms is in the range of 10 to 30 Å. The experimental papers[^27^, ^28^] are not very specific about the time resolution of the STM images on which the analysis of the In atom distribution is based, but these given distances are consistent with the experimental observations. In order to model the long times of inactivity of the In atoms, the experimental vacancy concentration of 10^−9^ needs to be realized in the simulations, but then the pairing of the vacancy with an In atom would be so rare that no proper statistics could be obtained within a reasonable simulation time.

## 3 Conclusions

In order to analyse the stability of surface alloys as a function of temperature and time, first principles kMC simulations have been employed modelling the vacancy mediated diffusion in PtAu/Au(111), PtRu/Ru(0001), AgPd/(Pd111) and InCu/Cu(001) single‐atom surface alloys. The energies barriers entering the kMC simulations via transition state theory have been derived from periodic DFT calculations. The migration of the foreign atom in the host metal can only proceed when the vacancy and the foreign atom become nearest neighbors and thus exchange places. Depending on the specific combination of foreign atom and host metal, the barriers for the exchange of the vacancy and the foreign atom range from 0.1 eV to 1.4 eV which leads to the fact that diffusion of the foreign atom is possible at room temperature or only at temperature above 1000 K. The apparent activation barrier is increased with respect to the simple exchange mechanism between vacancy and foreign atom, but only to a value in the range of the other diffusion barriers that contribute to the movement of the foreign atom. If the barrier for the exchange between vacancy and the foreign atom is signficantly larger than the vacancy diffusion barrier in the host metal, as in the system PtAu/Au(111), then the foreign atom will practically become immobile even at high temperatures as the vacancy will rather switch places with the atoms of the host metal than with the foreign atom. Finally, our kMC calculations confirmed that the apparent multi‐lattice‐spacing jumps in the system InCu/Cu(001) observed in STM experiments are a consequence of the low temporal resolution of the STM imaging.

## Conflict of interest

The authors declare no conflict of interest.

## Supporting information

As a service to our authors and readers, this journal provides supporting information supplied by the authors. Such materials are peer reviewed and may be re‐organized for online delivery, but are not copy‐edited or typeset. Technical support issues arising from supporting information (other than missing files) should be addressed to the authors.

Supplementary
