# Supplementary material for: Vacancy assisted diffusion on single‐atom surface alloys
Source: Chemphyschem. 2020 Dec 3;22(1):29–39. doi: 10.1002/cphc.202000838 (PMC7839753; doi:10.1002/cphc.202000838)
Supplement: Supplementary file 1 — Supplementary [file CPHC-22-29-s001.pdf]

Author Contributions

D.M. Investigation:Lead; Project administration:Supporting; Validation:Equal; Writing – original draft:Lead; Writing – review & editing:Equal
A.G. Conceptualization:Lead; Formal analysis:Equal; Funding acquisition:Lead; Project administration:Lead; Supervision:Lead; Validation:Equal; Writing – review & editing:Equal
